# Horizontal transfer of tRNA genes to mitochondrial plasmids facilitates gene loss from fungal mitochondrial DNA

**DOI:** 10.1007/s00294-022-01259-7

**Published:** 2022-11-30

**Authors:** Mathijs Nieuwenhuis, Jeroen Groeneveld, Duur K. Aanen

**Affiliations:** Laboratory of Genetics, Wageningen University & Research, Droevendaalsesteeg 1, 6708 PB Wageningen, The Netherlands

**Keywords:** Fungi, mtDNA, Mitochondrial plasmids, Horizontal gene transfer, Phylogenetics, Lyophyllaceae

## Abstract

Fungal and plant mitochondria are known to exchange DNA with retroviral plasmids. Transfer of plasmid DNA to the organellar genome is best known and occurs through wholesale insertion of the plasmid. Less well known is the transfer of organellar DNA to plasmids, in particular tRNA genes. Presently, it is unknown whether fungal plasmids can adopt mitochondrial functions such as tRNA production through horizontal gene transfer. In this paper, we studied the exchange of DNA between fungal linear plasmids and fungal mtDNA, mainly focusing on the basidiomycete family Lyophyllaceae. We report at least six independent transfers of complete tRNA genes to fungal plasmids. Furthermore, we discovered two independent cases of loss of a tRNA gene from a fungal mitochondrial genome following transfer of such a gene to a linear mitochondrial plasmid. We propose that loss of a tRNA gene from mtDNA following its transfer to a plasmid creates a mutualistic dependency of the host mtDNA on the plasmid. We also find that tRNA genes transferred to plasmids encode codons that occur at the lowest frequency in the host mitochondrial genomes, possibly due to a higher number of unused transcripts. We discuss the potential consequences of mtDNA transfer to plasmids for both the host mtDNA and the plasmid.

## Introduction

Mitochondria of plants and fungi are frequently hosts to retroviral plasmids. These plasmids can insert themselves wholly or partially in their host mitochondrial DNA (mtDNA). In some fungi, the insertions of plasmids in mitochondrial DNA (mtDNA) are linked to phenotypic effects, most notably senescence in *Neurospora* and *Podospora* (Griffiths et al. 1990; Tudzynski et al. 1980; van Diepeningen et al. 2008; Maas et al. 2004, 2005). However, in most fungi, the presence and occasional mitochondrial insertion of these plasmids is assumed to be inconsequential for the fungal phenotype. Plasmids can infect new hosts through horizontal transfer (Kempken 1995) but are also occasionally removed from their hosts during vertical transfer (Van Der Gaag et al. 1998). To persist in a host population, the spread of plasmids via horizontal transmission must therefore offset the loss through vertical transmission. Despite their abundance in nature, little is known about the means by which these plasmids spread and persist in their host populations.

Mitochondrial plasmids can be divided into two classes based on their structure: circular and linear, with linear plasmids further subdivided into invertron and non-invertron-like types (Griffiths 1995). Circular plasmids appear to use a rudimentary process of reverse transcription to replicate themselves (Galligan and Kennell 2007; Kennell et al. 1994). Invertron-like plasmids encode a DNA and RNA polymerase in inverted orientation to each other, and feature terminal inverted repeats bound covalently to proteins at the 5’ end. Invertron plasmids are thought to replicate in similar fashion to adenoviruses and certain bacteriophages, which share a similar structure (Griffiths 1995; Kempken et al. 1989), and their replication involves a protein primer. For invertron plasmids, the proteins bound to the terminal repeats probably play a role in plasmid replication.

A limited number of studies report transfer of mtDNA sequences to a plasmid. In *Neurospora*, partial mtDNA sequences were detected in circular plasmids of the *Varkud* and *Mauriceville* types, in addition to inserts of other, linear plasmid types (R A Akins et al. 1989; Mohr et al. 2000). In the circular plasmid *Mauriceville*, Chiang and Lambowitz (Chiang and Lambowitz 1997) found that reverse transcriptase activity was initiated without the use of a primer (as is common in reverse transcriptases) at the 3’ end of a tRNA-like structure on the plasmid. The plasmid reverse transcriptase could also initiate transcription at the 3’ end of a tRNA gene, resulting in cDNA synthesis of that tRNA gene (Chiang and Lambowitz 1997). Kempken (Kempken 1989) reported a tRNA-like structure on a linear plasmid found in *Ascobolus immersus*.

To our knowledge, only one study has reported transfer of a mitochondrial tRNA gene to a linear plasmid. In maize, a mitochondrial tRNA gene was found in a non-invertron-type linear plasmid (Leon et al. 1989). In the wild maize relative *Teosinte*, this plasmid-encoded tRNA gene has coincided with a loss of that tRNA gene in the mtDNA. This suggests the plasmid may have adopted the tRNA function, assuming that the tRNA is still required for the mitochondrion to function. If a plant or fungus becomes dependent on a plasmid for the production of a mitochondrial tRNA, it would present an interesting and rapidly evolving case of genetic addiction (Kobayashi 2004).

Addiction to a linear mitochondrial plasmid may be an important mechanism for their continued persistence. By acquiring a function that is vital or at least beneficial to the host, a plasmid can increase its chances of vertical transmission. Furthermore, transfer of mitochondrial DNA to linear plasmids may provide insight into their replication process. We studied the DNA exchange between fungal mitochondria and their associated linear plasmids, focusing in particular on transfer of mtDNA to plasmids. We focused on fungi of the Lyophyllaceae family as a whole-genome sequencing data set generated for a phylogenetic study (Van de Peppel et al. 2021) was available to screen for plasmids, and several complete mitochondrial genomes for this family are available to check for mtDNA transfer (Nieuwenhuis et al. 2019). In addition, Nieuwenhuis et al. previously reported four linear mitochondrial plasmids and numerous mitochondrial plasmid inserts in their study, suggesting that species in this family are often associated with such plasmids.

## Materials and methods

### Samples and sequencing

We obtained sequencing data of 44 Lyophyllaceae strains, most of which represent distinct species, from the Sequence Read Archive (SRA) to screen for linear mitochondrial plasmids (Table 1). Furthermore, we sequenced whole genomic DNA of ten additional *Termitomyces* strains (K2P1, Mi1657, T8, T28, T40b, T50a, T60a, T61, T70a, T99). The protocols for strain cultivation, DNA isolation, and sequencing were all identical to those outlined in Van de Peppel et al. (2021). To estimate intraspecific variation of plasmids, one *Termitomyces* species was represented by multiple strains in our data set (“*Termitomyces cryptogamus*”, Van de Peppel (2022), J132/T132/T99/T8/T28/T61/T60a). Data consisted of paired-end Illumina reads (read length: 150 bp, insert size: 500 bp). We acquired 18 complete sequences from GenBank of known linear mitochondrial plasmids occurring in fungi (Table 1). We also included a plasmid sequence discovered in a sample of *Trametes versicolor* in our lab. All plasmid sequences we discovered in this study have been submitted to GenBank under the accession numbers MW874118–MW874172 (Table 1). Since the majority of plasmids used in this study were found in the Lyophyllaceae, we used 12 mitochondrial genomes of Lyophyllaceae (mostly *Termitomyces*) to analyse DNA transfer between mtDNA and plasmids (Nieuwenhuis et al. 2019).

**Table 1:** Linear mitochondrial plasmid sequences used in this study

Plasmid	GenBank accession number	Host species	SRA accession number	Source
*Podospora anserina pAL2-1*	X60707.1	*Podospora anserina*	n.a	(Hermanns and Osiewacz 1992)
*Blumeria graminis pBgh*	AY189817.1	*Blumeria graminis f.sp. hordei*	n.a	(Giese et al. 2003)
*Claviceps purpurea pClK1*	X15648.1	*Claviceps purpurea*	n.a	(Oeser and Tudzynski 1989)
*Fusarium proliferatum pFP1*	EF622512.1	*Fusarium proliferatum*	n.a	(Láday et al. 2008)
*Moniliophthora roreri pMR1*	HQ259116.1	*Moniliophthora roreri*	n.a	(Costa et al. 2012)
*Lentinula edodes pLE*	AB697990.1	*Lentinula edodes*	n.a	(Yang et al. 2017)
*Moniliophthora roreri pMR3*	HQ259118.1	*Moniliophthora roreri*	n.a	(Costa et al. 2012)
*Pleurotus ostreatus mlp2*	AF355103.1	*Pleurotus ostreatus*	n.a	(Kim et al. 2000)
*Flammulina velutipes pFV*	AB028633.1	*Flammulina velutipes*	n.a	(Nakai et al. 2000)
*Gelasinospora Gel-kalDNA*	L40494.1	*Gelasinospora* sp. G114	n.a	(Yuewang et al. 1996)
*Neurospora intermedia kalilo*	X52106.1	*Neurospora intermedia*	n.a	(Shiu-Shing Chan et al. 1991)
*Neurospora crassa maranhar*	X55361.1	*Neurospora crassa*	n.a	(Court and Bertrand 1992)
*Neurospora intermedia Harbin-3*	AF133505.1	*Neurospora intermedia*	n.a	(Xu et al. 1999)
*Flammulina velutipes pFV2*	AB028634.1	*Flammulina velutipes*	n.a	(Nakai et al. 2000)
*Hebeloma circinans pHC2*	Y11504.1	*Hebeloma circinans*	n.a	(Bai et al. 1997)
*Moniliophthora roreri pMR2*	HQ259117.1	*Moniliophthora roreri*	n.a	(Costa et al. 2012)
*Pleurotus ostreatus mlp1*	AF126285.1	*Pleurotus ostreatus*	n.a	Unpublished
*Agaricus bitorquis pAB*	X63075.1	*Agaricus bitorquis*	n.a	(Robison et al. 1991)
*Arthromyces claviformis pAC*	MW874131	*Arthromyces claviformis*	SRX10708852	
*Asterophora parasitica pAP*	MW874136	*Asterophora parasitica*	SRX4910414	
*Calocybe cyanea pCC*	MW874161	*Calocybe cyanea*	SRX10337368	
*Calocybe cyanea pCC2*	MW874124	*Calocybe cyanea*	SRX10337368	
*Calocybe gambosa pCG*	MW874162	*Calocybe gambosa*	n.a	
*Calocybe gangraenosa pCGr*	MW874123	*Calocybe gangraenosa*	SRX10337362	
*Lyophyllum semitale pLSe*	MW874135	*Lyophyllum semitale*	SRX10337363	
*Lyophyllum shimeji pLS*	MW874160	*Lyophyllum shimeji*	n.a.	
*Tephrocybe p9980TJB*	MW874118	*Tephrocybe* sp. P9980TJB	SRX10337360	
*Termitomyces bulborhizus pTB*	MW874159	*Termitomyces bulborhizus*	SRX10337358	
*Termitomyces bulborhizus pTB 2*	MW874132	*Termitomyces bulborhizus*	SRX10337358	
*Termitomyces DKA64 pDKA64 1*	MW874172	*Termitomyces* sp. DKA64	SRX10337366	
*Termitomyces DKA64 pDKA64 2*	MW874153	*Termitomyces* sp. DKA64	SRX10337366	
*Termitomyces DKA64 pDKA64 3*	MW874155	*Termitomyces* sp. DKA64	SRX10337366	
*Termitomyces DKA64 pDKA64 4*	MW874122	*Termitomyces* sp. DKA64	SRX10337366	
*Termitomyces J132 pJ132*	MW874144	*Termitomyces* sp. J132	SRX255527	
*Termitomyces K1Aa pK1Aa 1*	MW874149	*Termitomyces* sp. K1Aa	SRX10313007	
*Termitomyces K1Aa pK1Aa 2*	MW874169	*Termitomyces* sp. K1Aa	SRX10313007	
*Termitomyces K1Aa pK1Aa 3*	MW874137	*Termitomyces* sp. K1Aa	SRX10313007	
*Termitomyces K1Ac pK1Ac mitochondrion*	MW874150	*Termitomyces* sp. K1Ac	SRX10337355	
*Termitomyces K1Ag pK1Ag 1*	MW874133	*Termitomyces* sp. K1Ag	SRX10337354	
*Termitomyces K1Ag pK1Ag 2*	MW874138	*Termitomyces* sp. K1Ag	SRX10337354	
*Termitomyces K2P1 pK2P1*	MW874139	*Termitomyces* sp. K2P1	SRX10767240	
*Termitomyces Mi1657 pTMi1657*	MW874127	*Termitomyces* sp. Mi1657	SRX10767235	
*Termitomyces pTDKA19*	MW874119	*Termitomyces* sp. DKA19	SRX4910405	
*Termitomyces T108 pT108 1*	MW874151	*Termitomyces *sp. T108	SRX10337365	
*Termitomyces T123 pT123 1*	MW874120	*Termitomyces* sp. T123	SRX4910411	
*Termitomyces T123 pT123 2*	MW874130	*Termitomyces* sp. T123	SRX4910411	
*Termitomyces T123 pT123 3*	MW874154	*Termitomyces* sp. T123	SRX4910411	
*Termitomyces T123 pT123 4*	MW874158	*Termitomyces* sp. T123	SRX4910411	
*Termitomyces T123 pT123 5*	MW874140	*Termitomyces* sp. T123	SRX4910411	
*Termitomyces T13 pT13*	MW874128	*Termitomyces* sp. T13	SRX4910413	
*Termitomyces T132 pT132 1*	MW874146	*Termitomyces* sp. T132	SRX4910412	
*Termitomyces T132 pT132 2*	MW874165	*Termitomyces* sp. T132	SRX4910412	
*Termitomyces T28 pT28 1*	MW874163	*Termitomyces* sp. T28	SRX10474844	
*Termitomyces T28 pT28 3*	MW874141	*Termitomyces* sp. T28	SRX10474844	
*Termitomyces T32 pT32*	MW874134	*Termitomyces* sp. T32	SRX4910409	
*Termitomyces T40b pT40b 1*	MW874125	*Termitomyces* sp. T40b	SRX10767241	
*Termitomyces T50a pT50a*	MW874126	*Termitomyces* sp. T50a	SRX10767238	
*Termitomyces T60a pT60a 1*	MW874143	*Termitomyces* sp. T60a	SRX10474845	
*Termitomyces T60a pT60a 2*	MW874166	*Termitomyces* sp. T60a	SRX10474845	
*Termitomyces T61 pT61 1*	MW874142	*Termitomyces* sp. T61	SRX10474846	
*Termitomyces T61 pT61 2*	MW874164	*Termitomyces* sp. T61	SRX10474846	
*Termitomyces T70a pT70a 1*	MW874171	*Termitomyces* sp. T70a	SRX10337364	
*Termitomyces T70a pT70a 2*	MW874129	*Termitomyces* sp. T70a	SRX10337364	
*Termitomyces T70a pT70a 3*	MW874152	*Termitomyces* sp. T70a	SRX10337364	
*Termitomyces T73sscA pT73sscA*	MW874148	*Termitomyces* sp. T73sscA	SRX10313000	
*Termitomyces T8 pT8 1*	MW874157	*Termitomyces* sp. T8	SRX10474843	
*Termitomyces T8 pT8 2*	MW874168	*Termitomyces* sp. T8	SRX10474843	
*Termitomyces T8 pT8 3*	MW874147	*Termitomyces* sp. T8	SRX10474843	
*Termitomyces T99 pT99 1*	MW874167	*Termitomyces* sp. T99	SRX10767243	
*Termitomyces T99 pT99 2*	MW874145	*Termitomyces* sp. T99	SRX10767243	
*Termitomyces titanicus pTT*	MW874170	*Termitomyces titanicus*	SRX10337357	
*Termitomyces titanicus pTT mitochondrion*	MW874156	*Termitomyces titanicus*	SRX10337357	
*Trametes versicolor pTV*	MW874121	*Trametes versicolor*	n.a	

### Assembly and plasmid detection

We assembled paired-end Illumina sequencing reads using SPAdes version 3.5.0 with default settings (Bankevich et al. 2012). We considered contigs potentially representing linear plasmids using their characteristic invertron-like structure. Contigs were sorted according to length and all contigs between 5 and 15 kb in size (all known invertron plasmids fall into this range) had their open-reading frames predicted using the mold mitochondrial code. Any contigs with two large (1 kb +) open-reading frames facing each other were considered potential plasmids. The two open-reading frames were then checked for homology to known plasmids using BLASTp on the NCBI web server with default settings (Expect threshold: 0.05; Word size: 6; Matrix: BLOSUM62; Gap Costs: 11/1; Conditional compositional score matrix adjustment; Filter low complexity regions) to verify they were plasmid-associated RNA and DNA polymerases. If so, the contig was considered to represent an invertron plasmid. After compiling all plasmids, we used BLASTn on the NCBI web server using default settings (Expect threshold: 0.05; Word size: 11; Match/Mismatch scores: 2/− 3; Gap costs: 5/2; Filter low complexity regions) to screen all assemblies again to search for plasmid sequences that perhaps had not been assembled contiguously and thereby had escaped detection, for example where the polymerases were split across two contigs.

Most plasmids gathered in this way most likely represent autonomous, non-inserted plasmids as they were capped on each end by an inverted repeat and their contig did not extend into a flanking region of mtDNA. Generally, inserted plasmids degenerate over evolutionary time and the polymerase coding sequences break down through stop codon accumulation. However, it is possible for a relatively recent plasmid insertion to be mistaken for an autonomous plasmid. In three *Termitomyces* samples (*T. titanicus, T. bulborhizus,* and *T. sp.* K1Ac), we found near-intact mitochondrial plasmid inserts. These could be distinguished from autonomous plasmids based on extended flanking regions beyond the terminal inverted repeats that shared homology with mtDNA. This shows that even recent insertions of complete plasmid sequences may be distinguished from autonomous copies by the flanking regions. We included these inserted plasmids in the phylogeny as the DNA and RNA polymerases were still intact.

### Sequence alignment and phylogenetic reconstruction

We aligned the translated amino acid sequences of the RNA and DNA polymerases separately using MAFFT (Katoh et al. 2002) with default parameters. The nucleotide sequences were translated to amino acids using the mold mitochondrial genetic code before alignment. We used the Gblocks online server (http://molevol.cmima.csic.es/castresana/Gblocks_server.html, Gblocks version 0.91b) (Castresana 2000) to remove poorly aligned regions, using the following settings: ‘allow smaller final blocks’, ‘allow gap positions within final blocks’, and ‘allow less strict flanking positions’. We then concatenated the trimmed alignments of both polymerases.

We used IQ-TREE for phylogenetic reconstruction (Nguyen et al. 2015). ModelFinder (Kalyaanamoorthy et al. 2017) found the best-fitting model for both the DNA and RNA polymerase alignment to be LG + F + I + G4. We ran IQ-TREE including 10,000 ultrafast bootstraps (Hoang et al. 2018). Nodes with bootstrap support values below 95 were collapsed into polytomies. We included a midpoint root as an approximate rooting, since there is no known closely related sister group to these linear plasmids. Some plant plasmids are suspected to form a sister group, but that relationship was not significantly supported in our trial phylogeny (data not shown). Previous studies have used phage polymerases as outgroup (Andrade and Góes-Neto 2015), but we considered these too divergent to be informative.

### Detection of DNA exchange between mtDNA and plasmid

We performed reciprocal BLASTn searches of curated, complete mitochondrial genomes of twelve Lyophyllaceae species (Nieuwenhuis et al. 2019) to all plasmid sequences. We considered hits significant if they exceeded 50 bp in length and had an E-value < 0.001. To test for loss of genomic tRNA gene copies, we performed BLASTn searches of draft genome assemblies of all strains of *T.* sp. T132 (T132/J132/T99/T61/T60a/T28/T8) (including nuclear, mitochondrial, and plasmid-derived contigs) using the mitochondrial tRNA-Arg (TCG) gene sequence of the sister species *T. sp.* T123 as query. We also performed this analysis for the strains T. sp. T13/T50a/T40b using the mitochondrial tRNA-Cys gene sequence of strain T. sp. Mi1657.

To verify tRNA sequences and examine the effect of mutations on their secondary structure, we used the tRNA-Scan web server (Chan and Lowe 2019). We set sequence source to ‘Other Mitochondrial’ and genetic code to ‘Mold & Protozoan Mito’, with all other settings as default.

## Results

Using a combined sequence similarity search and structural identification of contigs (see Materials and Methods for details), we collected 55 plasmid sequences from 54 whole-genome sequence assemblies of different fungal strains (all from the family Lyophyllaceae). With an additional 18 plasmid sequences found on GenBank, our final data set comprised 73 plasmid sequences from 46 different host strains. The highest number of different plasmid strains found in a single host was five for *Termitomyces* sp. T123. We observed intraspecific variation of plasmid presence among host species for which multiple strains were analysed: we detected up to three different types of plasmid in *Termitomyces cryptogamus* (Van de Peppel 2022), ranging from one in strain J132, to three in strain T8, with another two plasmid types in the five other strains of this species.

### Phylogeny

**Fig. 1:**
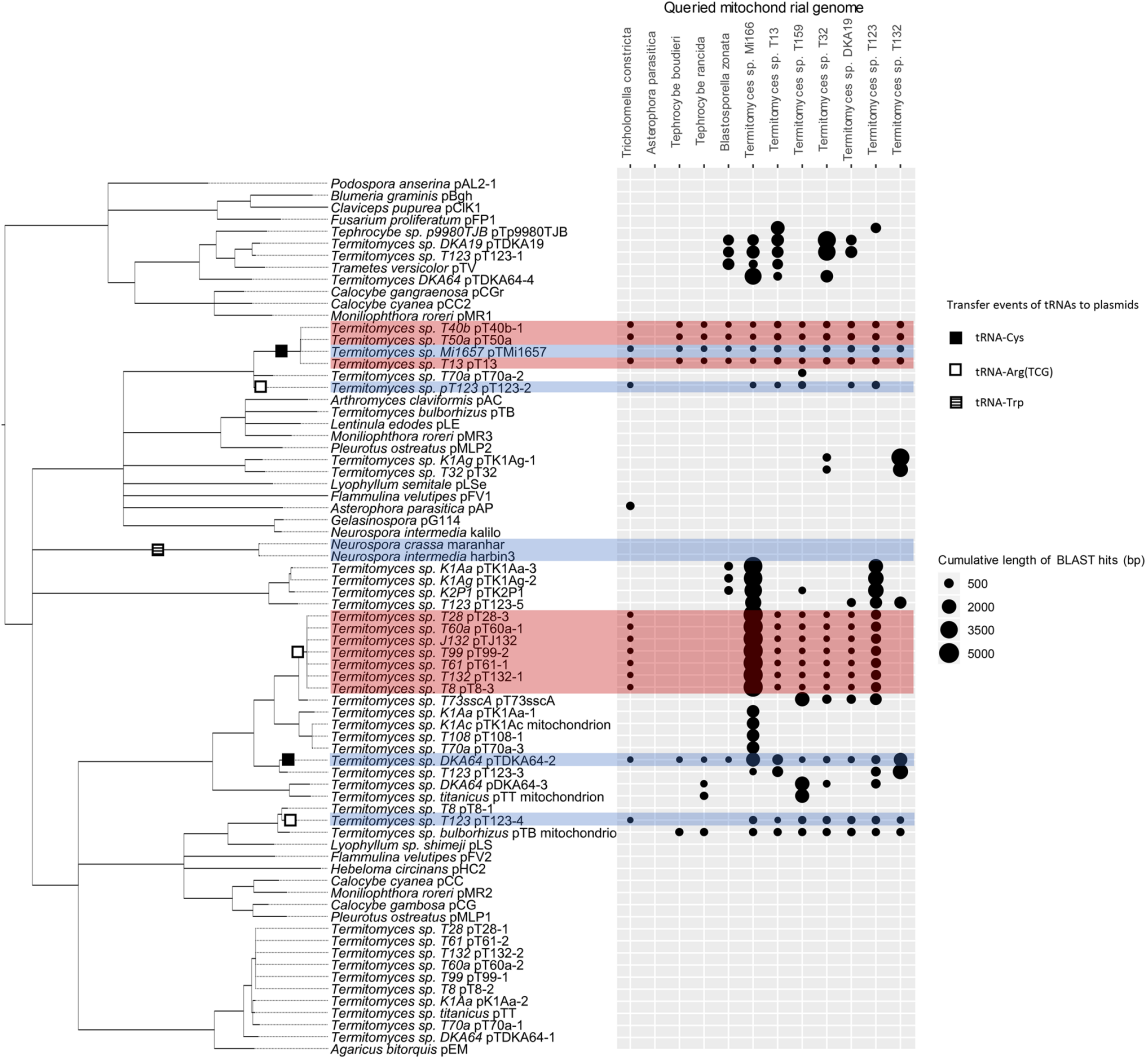
Midpoint-rooted maximum-likelihood phylogeny of fungal linear plasmids. Nodes with less than 95 ultrafast bootstrap support values were collapsed. Plasmids are named after the host species or strain in which they are found. In strains with multiple plasmids, the name is extended with a number to differentiate them. Plasmids that were found as intact mitochondrial insertions are indicated with the word ‘mitochondrion’. The graph on the right indicates the presence and cumulative length of homologous regions found between each plasmid and 12 mitochondrial genomes of the Lyophyllaceae. Plasmids with adopted mitochondrial tRNA genes are color-coded in red (in case the same tRNA gene was lost in the host mtDNA) and blue (in case the same tRNA gene is still present in the host mtDNA). Inferred independent transfer events of tRNA genes to plasmids are indicated by squares on the phylogeny (black for tRNA-Arg (TCG), white for tRNA-Cys, and striped for tRNA-Trp)

As expected based on previous research (Andrade and Góes-Neto 2015; Griffiths 1995; Van Der Gaag et al. 1998), the plasmid phylogeny is incongruent with the host fungal phylogeny (Fig. 1), with many clades consisting of plasmids from ascomycete and basidiomycete hosts, for example. Deep nodes have poor support and are mostly collapsed to polytomies, due to presumed high mutation rates and short alignment length resulting in overall poor homology. However, we obtained strong support for several clades of relatively closely related plasmids. Many of these clades consist of a mix of Lyophyllaceae-associated plasmids and plasmids of more distantly related hosts.

### Plasmid–mtDNA integrations

Comparison using BLAST of mitochondrial DNA to all plasmid sequences revealed numerous homologous sequences between plasmids and mtDNA (Fig. 1). In most cases, these potential inserts were small and highly degraded, with the polymerase genes interspersed with numerous stop codons. In host mtDNA infected with autonomous plasmids, inserts often do not share homology with those autonomous plasmids, suggesting that the inserts derive from plasmids that have since disappeared from that host. Inserts were also often found in species for which we found no autonomous plasmids, again indicating past presence of plasmids.

### Transfer of mtDNA to plasmid

**Fig. 2:**
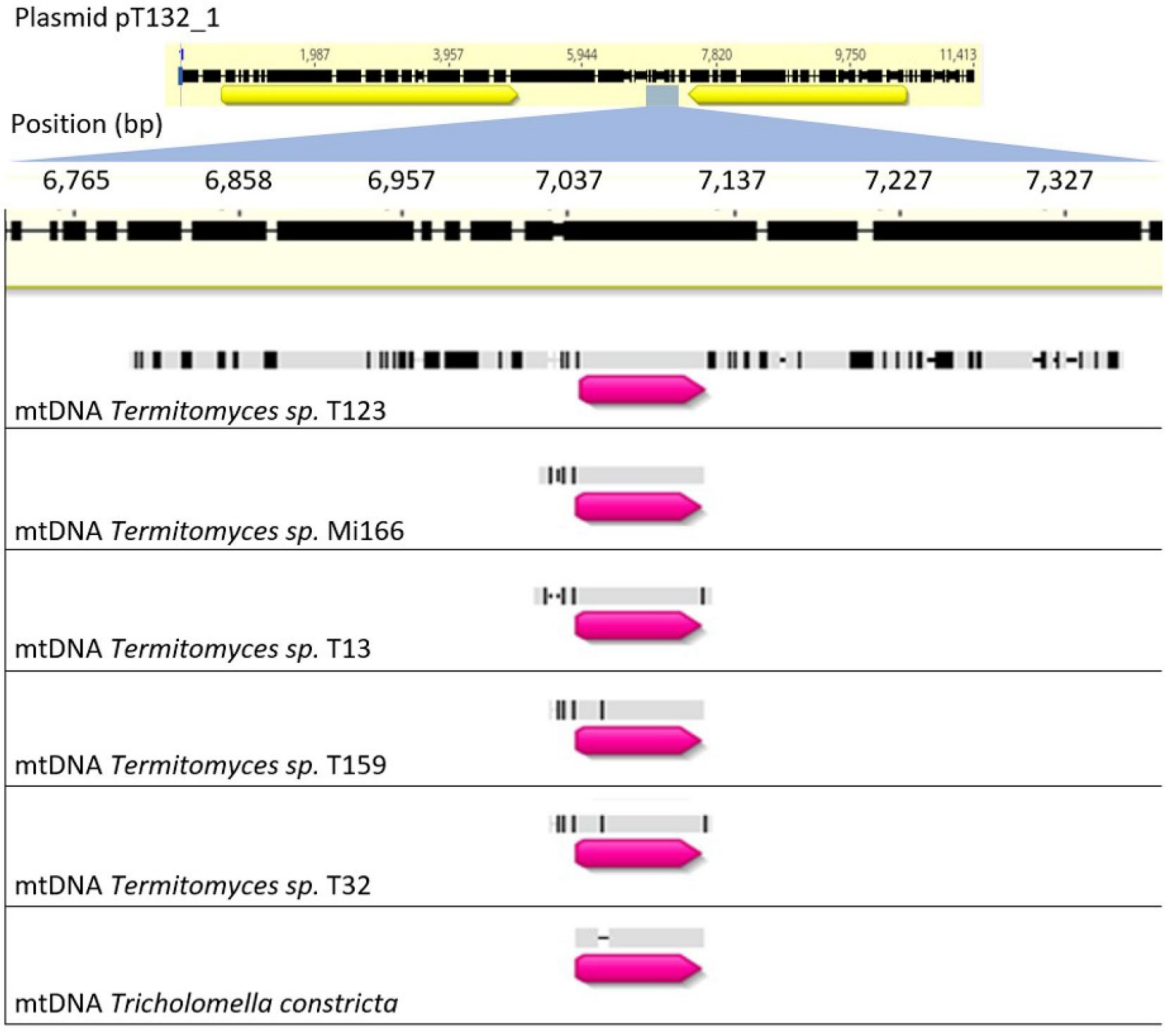
Homology of tRNA-Arg (TCG) of several Lyophyllaceae mitochondrial genomes and a region of plasmid pT132-1 detected by Megablast. A roughly 600 base pair section of pT132-1 located between the DNA and RNA polymerase is shown (black bar, top). Below are shown homologous regions from the mtDNA of several Lyophyllaceae species (grey bars, black sections indicate sequence divergence). The location and relative size of tRNA-Arg (TCG) for each species is indicated by the pink arrow. While most species only show a significant match for the exact location of tRNA-Arg (TCG), *Termitomyces sp.* T123 shares significant homology with the plasmid for the flanking regions as well (up to around 300 base pairs upstream and downstream). Note that the host species carrying the plasmid, *Termitomyces sp.* T132, showed no significant homology between the plasmid and its mtDNA

We observe at least six independent adoption events of tRNA genes by plasmids, once in *Neurospora* and five times in *Termitomyces* (Fig. 1). In plasmids pT132-1/pJ132/pT99-2/pT8-3/pT28-3/pT60a-1/pT61-1 (all from the species *Termitomyces cryptogamus*), we detected a sequence homologous to tRNA-Arg (TCG) from mitochondrial genomes of *Termitomyces* and close relatives (Fig. 2). In particular, flanking regions of this plasmid sequence matched flanking regions of tRNA-Arg (TCG) in the mtDNA of the sister group of *T. cryptogamus*, *T.* sp. T123. Furthermore, in the mitochondrion of *T.* sp. T132, this mtDNA region lacks the tRNA-Arg (TCG) gene as well as homologous sequences of the flanking regions. This suggests that in the ancestor of *T. cryptogamus*, this part of mtDNA was copied to a plasmid, after which the mitochondrial copy was lost. The draft genome assembly of *T.* sp T132 (Van de Peppel et al. 2021) contained only one copy of this tRNA gene, which was the plasmid-bound copy. Although we found many mismatches between the plasmid pT132-1 and the mtDNA of *T.* sp. T123 within the flanking regions, we found no mutations in the tRNA itself. As we do not know the exact timing of the tRNA transfer to the plasmid following the divergence of *T.* sp. T132 and *T.* sp. T123, it is unclear if these mutations mostly occurred in the host mtDNA prior to transfer, or in the plasmid after transfer.

*Termitomyces sp.* T123 is associated with five different linear plasmids, all of which have been detected in the same isolate. Two of these plasmids contain a copy of the tRNA-Arg (TCG) gene but have acquired it independently from each other and from the plasmid found in T. *cryptogamus* (Fig. 1). The same tRNA-Arg (TCG) gene is also still found in the mtDNA of *T.* sp. T123, so the host mtDNA is not dependent on the plasmid copies for this anticodon. In plasmid pT123-2, the tRNA-Arg (TCG) carries mutations that alter its secondary structure according to a structural prediction made using tRNA-Scan. In plasmid pT123-4, no mutations have occurred in the tRNA-Arg (TCG) gene relative to its host mitochondrial counterpart, but whether this is due to selection pressure or due to this copy being a recent acquisition is unclear. Plasmid pT123-4 also contains a sequence matching the first 48 amino acids of the *nad2* gene, with several mutations at the 5′-end.

Plasmids pT50a/pT40b/pTMi1657/pT13 possess a copy of tRNA-Cys. This gene is missing in the mtDNA of *Termitomyces* sp. T13/sp. T50a/sp. T40b, indicating another transfer of a tRNA followed by mitochondrial loss in some strains carrying this plasmid but not in all. In *Termitomyces* sp. DKA64, we also found a plasmid with a copy of tRNA-Cys, with no loss of this tRNA in the mtDNA of this strain. As this plasmid is not closely related to the other four, this likely represents an independent transfer event.

We also found that the maranhar plasmid associated with *Neurospora crassa* contained a tRNA-Trp gene. A copy of this same tRNA-Trp gene has been detected in the *Neurospora*-associated circular plasmids, *Varkud* and *Mauriceville* (Akins et al. 1989).

For strains in which we observed loss of a mitochondrial tRNA gene copy, we used BLAST to search for a copy of this tRNA gene in the nuclear genome assembly, using the plasmid-borne copy as a reference. We found no sequences matching the tRNA gene sequence in any contigs apart from the plasmid sequence itself.

### Codon frequency of captured tRNAs

**Fig. 3:**
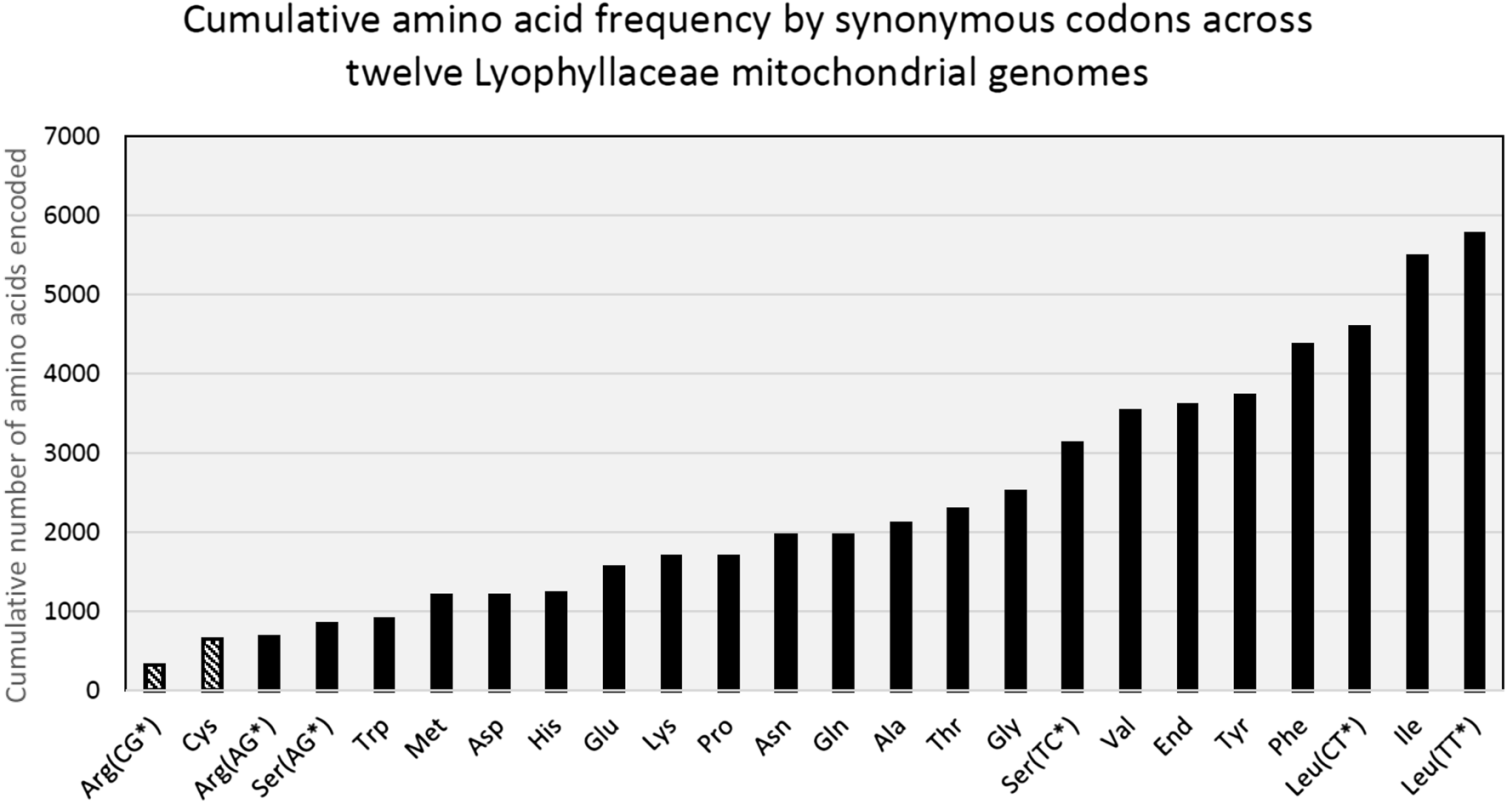
Cumulative amino acid frequency of the fourteen principal mitochondrial genes (*atp6, atp8, atp9, cob, cox1, cox2, cox3, nad1, nad2, nad3, nad4, nad4L, nad5, nad6*) for 12 Lyophyllaceae mtDNAs. We grouped amino acids by synonymous codons, which correspond to the anticodons carried by mitochondrial tRNAs. The amino acid codons for which we observe transfer of the corresponding tRNA gene to a mitochondrial plasmid (in the Lyophyllaceae) are shown with diagonal stripes

Of the 24 different tRNA genes present in almost all fungal mtDNA, we found only three in plasmid sequences: three cases of tRNA-Arg (TCG), two of tRNA-Cys and one tRNA-Trp (in *Neurospora*). We hypothesized that the probability of adoption of a tRNA gene by a plasmid may depend on the frequency of the corresponding codon found in the mitochondrial genes. We therefore calculated the average codon use for the 12 Lyophyllaceae mitochondrial genomes (Fig. 3). We found that the tRNA genes transferred to plasmids in Lyophyllaceae (tRNA-Arg (TCG) and tRNA-Cys) rank the lowest in terms of their corresponding codon use in mitochondrial genes.

## Discussion

### Plasmid diversity and evolution

Invertron plasmids are thought to be the most abundant group of mitochondrial plasmids in fungi (Griffiths 1995). Of the 54 Lyophyllaceae strains sampled for this study, over half (28) had at least one associated plasmid strain. Studies in *Podospora* (Van Der Gaag et al. 1998) and *Neurospora* (Arganoza et al. 1994; Maas et al. 2005) have previously shown intraspecific variation in plasmid presence of fungal isolates. Consistent with this, we found intraspecific variation in plasmid infection for the seven strains we analysed of one *Termitomyces* species, *T. cryptogamus* (*T.* sp. J132, sp. T132, sp. T8, etc.), which has been shown to be a single biological species (De Fine Licht et al. 2006; Nobre et al. 2014). As such, it is likely that in at least some of the species for which our sequenced strains showed no plasmids in this study, strains in the wild exist that do have plasmids.

Our phylogenetic tree (Fig. 1) combines the 54 newly discovered plasmids from the Lyophyllaceae with 18 that were previously reported in other fungi, and shows significant support for clades incongruent with the host–fungal phylogeny. Several clades contain a mixture of plasmids derived from ascomycetes and basidiomycetes. The majority of plasmids analysed in this study were found in the genus *Termitomyces*, and many of these cluster into different clades with plasmids from other fungi. These incongruencies with the host phylogeny can partly be explained by a long history of differential loss of plasmids among hosts. However, considering the fast mutation rate of these plasmids (Warren et al. 2016) and the relatively low genetic divergence between plasmids of different host genera, horizontal transmission of plasmids between different fungal species probably plays a significant part as well.

A couple of plasmids we discovered in two *Calocybe* species (pCGr and pCC2) did not conform to the expected invertron structure, with the DNA polymerase and RNA polymerase genes positioned on the same strand. Both plasmids were still capped on each end by terminal inverted repeats. In our phylogenetic reconstruction (Fig. 1), these plasmids form a clade with one of the plasmids associated with *Moniliophthora roreri* (pMR1), which also has a non-invertron structure (Costa et al. 2012).

### Plasmid insertions in mtDNA

Sequence comparison of 12 Lyophyllaceae mitochondrial genomes to our plasmid data set revealed numerous and sizeable plasmid insertions in mtDNA, even in species for which no autonomous plasmid was detected (Fig. 1). Furthermore, in species with known plasmid infections, mitochondrial inserts were often not homologous to the autonomous plasmid. Since most plasmid inserts were fragmented and degraded, these findings suggest most inserts represent ancestral insertion events that occurred between the ancestral mtDNA and an autonomous plasmid that in many cases no longer occurs in the isolate.

### Transfer of mtDNA to plasmids

Transfer of mitochondrial DNA to linear and circular plasmids has previously been described in fungi (Akins et al*.* 1986, Akins et al. 1989, Kempken 1989, Mohr et al. 2000) and in plants (Leon et al. 1989). In circular plasmids, such transfers may occur by the same reverse transcription process that drives plasmid replication (Chiang Lambowitz 1997). The transfers we identified in invertron plasmids may also result from replication errors by the plasmid, or a more general process of insertion. The replication process of invertron plasmids is thought to involve a protein primer, using the proteins covalently bound to the terminal inverted repeats. This differs significantly from circular plasmids, which are thought to be able to replicate without a primer but initiate close to a tRNA-like structure.

We found at least two separate occasions of mitochondrial loss of a tRNA gene following the transfer of a copy of that gene to a plasmid (Fig. 1). Leon et al*.* found that in the maize relative *Teosinte*, the transfer of a tRNA-Trp gene coincided with its loss in the mtDNA. Warren et al. (2016) suggested that transfer of mtDNA to plasmids contributes to accelerated sequence evolution of such genes due to the higher mutation rate of plasmids. Our findings of conserved tRNA sequences in plasmids show that such sequences may in fact remain relatively free from mutations when the mitochondrial copy is lost and the transferred sequence encodes an essential function.

Mitochondria import many tRNAs from the nucleus, which could be an alternative explanation for mitochondrial gene loss from mtDNA. However, the sudden loss of tRNAs co-occurring with the appearance of a functional copy in a mitochondrial plasmid that appears to be fixed in the population suggests the tRNA function has transferred to the plasmid and not to the nucleus. In the strains that showed loss of a mitochondrial tRNA gene, we found no evidence of gene copies in the nuclear genome assemblies for these lost mitochondrial tRNA genes. In addition, we only observe loss of mitochondrial tRNA genes in Lyophyllaceae mtDNA when a copy of that tRNA gene is present on an autonomous plasmid. If tRNA transcripts were imported from the nucleus, loss of a mitochondrial tRNA would occasionally be expected in the absence of a plasmid copy. Yet, in all 12 complete mitochondrial genome sequences of Lyophyllaceae species available to us, the only two instances of mitochondrial tRNA gene loss occurred when the lost tRNA gene had been transferred to a plasmid.

Ferandon et al. (Ferandon et al. 2008) reported a tRNA-Met gene located on a linear plasmid that had integrated in the mtDNA of its host, the fungus *Agrocybe agerita*, and suggested that the tRNA gene may have been captured by the plasmid prior to integration. Mitochondrial insertion of plasmids carrying mitochondrial tRNA genes or other mitochondrial genes poses an interesting evolutionary process. First of all, plasmids can be horizontally transferred between different species, creating the potential for indirect horizontal transfer of mtDNA between these species. Second, following mitochondrial loss of a tRNA gene that transferred to a plasmid, the plasmid could reintroduce the tRNA gene to the mtDNA through insertion. This would likely alter the location of the tRNA gene in the mtDNA. It would also nullify any selective benefit the plasmid enjoyed from the transferred tRNA gene if the mtDNA is able to recover the tRNA function.

Plasmid pT123-4 also contains portions of nad2, a mitochondrial protein-coding gene. This shows plasmids are capable of capturing other parts of mtDNA besides tRNA genes, but since captured sequences are generally limited in size, capturing a complete functional gene may be restricted to tRNA genes and small genes like *atp8*, *atp9*, and *nad4L*.

### All tRNAs found in plasmids code for low-frequency codons

We observe at least five independent acquisitions of tRNA genes by plasmids in the genus *Termitomyces*, twice of tRNA-Cys and three times of tRNA-Arg (TCG). Why do we only observe transfer of these tRNA genes and no others? It is possible that the capture of mitochondrial tRNAs by plasmids is dependent on certain sequence motifs found only nearby these tRNA genes. However, the intergenic sequences are not well conserved between *Termitomyces* species making this unlikely. Looking at the codon usage of mitochondrial genes (Fig. 3), tRNA-Cys and tRNA-Arg (TCG) encode anticodons for the least frequently used of all codons in the corresponding mtDNA. If the production of tRNA transcripts by plasmids is lower than that of mitochondrial tRNA genes (as they are not adapted for their transcription), it is possible that plasmids can only meet the mitochondrial demand of rare anticodons. Such captured tRNA genes can then be selectively beneficial if the mitochondrial copy is lost or mitochondrial transcription of that tRNA gene is otherwise inhibited. However, this would not explain why we also find only low-frequency codon-coding tRNA genes in plasmids when the host mtDNA has not lost its own copy. Fungal mtDNA is thought to be generally transcribed in large polycistronic transcripts (Kolondra et al. 2015), which might result in roughly equal transcription rates for tRNA genes. It is possible that low-frequency codons have more unused transcripts available for integration by a plasmid, increasing their chance of transfer to a plasmid.

Capture of tRNA genes coding for codons commonly used in the host mtDNA was shown by Akins et al. (Akins et al. 1989) in *Neurospora crassa,* where circular plasmids were found containing tRNA-Trp, tRNA-Val, and tRNA-Gly. These three tRNA genes code for codons that appear in fungal mtDNA at relatively high frequencies (Fig. 3). This shows that it is possible for plasmids to adopt high-demand tRNA genes, but for some reason, we did not observe any such cases in linear plasmids.

## Conclusions

We recovered 55 mitochondrial linear plasmid sequences from the Lyophyllaceae and analysed DNA transfer between these plasmids and their host mtDNA. We observed several independent transfers of complete mitochondrial tRNA genes to linear plasmids. In two cases, this transfer coincided with a loss of the tRNA gene in the host mtDNA, potentially resulting in genetic addiction of the host to the plasmid. We also show that tRNA genes transferred to plasmids tend to code for rare mitochondrial codons. Our results show that the interaction between fungal mtDNA and linear mitochondrial plasmids is much more dynamic than previously thought. The potential addiction of mtDNA to a plasmid through the exchange of a tRNA gene shows how a mutualistic association can arise abruptly between a selfish genetic element and its host genome.

## Data Availability

Sequence data generated for this article are available from GenBank under the accession numbers MW874118–MW874172.
